# Therapists’ and patients’ experiences with electronic patient-reported outcome measures (PROMs) and patient-reported experience measures (PREMs) before and during treatment in community mental health services: a qualitative study in Norway

**DOI:** 10.1186/s12888-025-07541-5

**Published:** 2025-11-13

**Authors:** Eirik Roos, Thomas Hugaas Molden, Martin Schevik Lindberg, Heidi Westerlund, Karl Johan Johansen, Audun Havnen

**Affiliations:** 1 Health and Welfare, Trondheim Municipality, P. O. 2300, Trondheim, 7004 Norway; 2 Department of Psychology, Norwegian University of Science and Technology, Trondheim, 7491 Norway; 3 KBT Vocational College, Trondheim, 7031 Norway; 4 Division of Psychiatry, Nidaros Community Mental Health Care, St. Olav’s University Hospital, Trondheim, 7040 Norway

**Keywords:** Community mental health, Experience with PROMs/PREMs, Qualitative study

## Abstract

**Background:**

The implementation of electronic patient-reported outcome measures (PROMs) and patient-reported experience measures (PREMs) in community mental healthcare is a complex experience for both therapists and patients. Barriers need to be identified and overcome. The aims of this study were to examine how therapists and patients experienced the implementation of digital PROMs and PREMs in mental healthcare, and to identify those factors that promote positive experiences with mental health treatment supported by PROMs and PREMs.

**Methods:**

In this descriptive qualitative study, we conducted focus group interviews with 19 therapists and individual interviews with six patients who had received low-intensity cognitive behavioural therapy interventions. All informants were interviewed about their experiences following the implementation of self-reported electronic measures in an outpatient treatment facility. The central question posed to all informants concerned their experience with electronic questionnaires. The interviews were analysed with a thematic approach using systematic text condensation.

**Results:**

Both therapists and patients experienced initial barriers in the form of a lack of information and guidance about answering questionnaires. Both the therapists and patients experienced improved insight into symptoms and challenges. This enhanced awareness led to the patient being more actively involved in the formulation of objectives in the treatment process. Patients reported that the implementation of PROMs and PREMs led to better informed therapists who listened more actively and asked relevant questions about adherence and current struggles in daily life.

**Conclusion:**

Overall, patients with mental health problems need practical guidance on how to complete self-report questionnaires. By providing their own feedback, patients’ self-awareness of their symptoms and challenges was strengthened, thereby facilitating discussions on how to best align treatment goals to treatment needs. Several patients highlighted the importance for PREMs of the therapists’ attitudes and skills. The findings of this qualitative study suggest that both PROMs and PREMs can be implemented successfully in routine mental healthcare when care is taken to identify and overcome barriers.

**Supplementary Information:**

The online version contains supplementary material available at 10.1186/s12888-025-07541-5.

## Background

Although mental health research has seen the extensive use of clinician-administered assessments [1, 2], the outcomes and preferences reported by patients themselves are viewed as important supplements to these clinician assessments [3]. At the individual level, patient-reported outcomes might facilitate greater patient involvement during treatment, and at the system level, services can be further developed to better fit patients’ needs [4]. Enabling patients to report clinical outcomes and processes has also attracted greater interest, and there is increased concern about the importance of systematically receiving feedback on patients’ experiences with the health services they have received [4–6].

Patient-reported outcome measures (PROMs) are standardised, validated instruments that are completed by patients to measure their health and well-being, especially to assess changes in outcomes after the implementation of a new intervention. Patient-reported experience measures (PREMs), on the other hand, are measures of a patient’s perceived experience of the healthcare received. PROMs include reporting patients’ own perspectives on their health status, whilst PREMs assess patients’ experiences of using health services and treatment [7–10]. PROMs and PREMs are increasingly used in research on many health conditions [11, 12], including electronically administered PROMs in mental healthcare [13–15]. Previous research showed that progress feedback can significantly improve treatment outcomes and reduces dropout [16, 17].

PROMs data are an important source of information regarding patient experiences and outcomes, and are highly relevant for clinical practice. Studies demonstrate that PROMs may lead to a range of positive outcomes such as improved clinician–patient communication [18]. The quality of mental healthcare services may be enhanced by using aggregated patient feedback to better tailor services to the needs of the patients [19]. A randomised trial of PROMs for monitoring outcomes for patients with depression in primary care in England revealed that the use of PROMs was associated with improved quality of life [20]. PROMs and PREMs are therefore valuable sources of information that may lead to better outcomes, improved healthcare, and enhanced decision-making in routine clinical care.

A meta-review identified distinct barriers that were experienced by therapists and service users when using PROMs. The therapists’ barriers were a perceived lack of resources to apply the PROMs, fear of critical evaluation of therapist skills and lack of administrative support [21]. Another review identified important steps that need to be taken to ensure the successful implementation of routine monitoring, such as sufficient training in the measures used and administrative support [14]. A study on the use of PROMS in cancer care suggested that barriers at the patient level included lack of time and difficulty using electronic assessment systems [22]. At the health professional level, barriers involved lack of time and limited knowledge regarding how to interpret and include PROM results in clinical practice. At the organisational level, barriers were related to difficulties in integrating PROMs in routine clinical practice, and technology was a limiting factor in establishing an effective infrastructure.

The Norwegian mental healthcare system is organised into two levels – community mental healthcare for patients with mild severity and specialist care for patients with moderate to severe severity. Recent years have seen an increase in the resources allocated to Norwegian community mental healthcare to improve access to low-intensity interventions for persons with mental problems [23]. In community mental healthcare, PROMS are just beginning to be implemented, and knowledge of how such systems are experienced by the users remains limited.

Quantitative surveys are prone to systematic bias if dissatisfied patients fail to complete posttreatment assessments, either because of a lack of satisfaction with the treatment or dissatisfaction with the system used for patient monitoring [13, 24]. There is therefore a need for qualitative research to investigate the perceived benefits and barriers reported by both the patients who complete the assessment and the therapists who use the assessment.

According to the consolidated framework for implementation research, qualitative research on implementing clinical PROM programmes at US hospitals has revealed both facilitators of and barriers to implementation success [25]. To ensure this success, the authors suggested the following key facilitators: allowing clinicians to select PROM measures and ensuring a user-friendly data platform; adapting data collection to the patient’s home environment; informing clinicians of the multifaceted use of PROM data for research and clinical care; implementing PROM education earlier in clinician training; and establishing speciality-diagnostic PROM implementation teams [25]. One previous qualitative study followed 26 professionals who had experience of how patient-reported measures (PRMs) can be used to improve person-centred coordinated care [26]. However, this study revealed that barriers to the use of PROMs were related to a lack of a whole-service approach to implementation.

Studies have illustrated how cultural aspects may influence PROMs/PREMs [27, 28], and the importance of conducting studies within different countries and cultural settings. Previous qualitative work has explored how therapists and patients experience the implementation of PROMs in Norwegian specialist mental healthcare [29, 30]. One study highlighted “shared needs” as a dominant domain in the material [29], where the perspectives of different stakeholders converged, thereby emphasising the importance of trust and openness in therapy. Hovland et al. [30] found that patients and therapists collaboratively shaped the meaning and relevance of the clinical feedback system (CFS) by interpreting its measures to reflect the unique patient–therapist relationship. Patients viewed the CFS as a valuable tool for highlighting important therapy-related issues, which helped them to become more self-aware and better prepared for sessions. However, there is a need for more qualitative studies in Norwegian community mental healthcare to increase knowledge of how patient feedback systems are perceived by patients and therapists.

In the present study, we aimed to investigate therapists’ and patients’ experiences with the implementation of PROMs and PREMs in a Norwegian community mental healthcare setting. Moreover, we aimed to identify those factors that promote positive experiences with treatment supported by PROMs and PREMs.

## Methods

### Participants

Nineteen therapists and six patients with mental health problems were recruited from a community mental health service and participated in this study. Characteristics of the informants are reported in Tables 1 and 2.

**Table 1:** Characteristics of the therapist informants (n = 19)

Variable	*n*	Interview group one	Interview group two	Interview group three
Male	3	-	-	3
Female	16	6	6	4
*Total*	*19*	*6*	*6*	*7*
Profession Nurse Social worker Occupational therapist	10 3 6	4 - 2	3 1 2	3 2 2

**Table 2:** Characteristics of the patient informants (n = 6)

Variable	*n*
Male Female	5 1
Mean age (SD)	28.5 (3.9)
Profession Student In paid work (part-time) + disability benefit* Disability benefit*	*n* 1 2 3

Note. *Disability benefit provides a secure income for those who have a permanently reduced earning capacity due to illness or injury

### Recruitment

To recruit therapist informants, the team leader for the services introduced the study both orally and by handing out invitation letters to eligible therapists. In the invitation letter, therapists were informed that they could choose between focus group or individual interviews. The team leader provided contact information for those therapists who wanted to participate to the first author (ER), who then contacted them and organised the focus group interviews at the informants’ workplace.

To recruit patient informants, therapists at the community mental health centre introduced the study both orally and by handing out invitation letters to eligible patients, who were already included in the research project “Systematic Quality Assurance of Primary and Specialist Mental Health Care” [23]. The invitation letter stated that the patients could choose between group or individual interviews. The therapists informed the first author (ER) by telephone about the patients who were willing to participate in the study. We aimed to recruit 10–15 patient informants.

The study was approved by the Regional Committee for Medical and Health Research Ethics (case number REK 2019/31836) and the Norwegian Centre for Research Data (case number 605327). In accordance with the Declaration of Helsinki, informed consent was obtained from the informants before the interviews were conducted, and confidentiality was assured. All participants were informed that they could withdraw their consent at any time. None of the participants withdrew their consent.

### Research team and reflexivity

ER (PhD) is a registered nurse with 30 years of experience in the municipality.

THM (PhD) is a sociologist who currently holds a position at Trondheim Municipality.

MSL is a clinical psychologist with 10 years of experience and is currently a PhD student at the Norwegian University of Science and Technology. EW has extensive experience of research involving user representatives, and holds a position at KBT Vocational College, which educates peer support workers. KJJ holds a position as principal of the KBT Vocational College. AH (PhD) is a clinical psychologist with 14 years of clinical experience, and holds a position of professor at the Norwegian University of Science and Technology.

The research group shares an interest in conducting research in naturalistic clinical settings. The members of the group have varying degrees of preexisting knowledge and experience in the field of study. ER has extensive experience of qualitative research in real-world clinical settings. EW is a peer support worker at KBT Vocational College, with an interest in promoting patients’ perspectives when implementing PROM/PREM processes in mental healthcare. KJJ has extensive experience in facilitating mental health peer support. AH has extensive experience of clinical effectiveness research. Several of the authors (ER, THM, HW and KJJ) have extensive experience of qualitative interviews with persons with mental health challenges. At the start of the study, all the researchers had several years of experience with working in the field of mental healthcare and research, with an interest in implementing PROMs/PREMs and conducting clinical research in mental healthcare. Neither the researchers nor the interviewers had established a relationship with any of the participants prior to the interviews.

### Setting

The study took place in a Norwegian city with approximately 200,000 inhabitants. By 2022, the municipality had 2,580 patients registered in community mental health services and had 283 full-time employees. Patients could self-refer to the community mental health centre or book an appointment by telephone without a referral from a general practitioner (GP). The patient informants were characterised by one of the following features:

1. recently developed mild to moderate health problems.
2. mental health problems in addition to more complex life challenges.
3. concurrent addiction and mental health problems.

### Data collection method

We followed the consolidated criteria for reporting qualitative studies (COREQ) [31] to ensure comprehensive reporting (Supplementary Material).

During all interviews, only the researchers and informants were present. All interviews were conducted in Norwegian and relevant quotes were translated to English for the current paper.

An interview guide developed by the authors (Supplementary Material, S1) was used in all therapist interviews. The interview guide was not pilot tested but was quality checked to ensure it was comprehensive and well understood after the first interview. The first (ER) and second (THM) authors conducted the therapist interviews. The questions were open-ended and order-flexible, which allowed the researcher to pursue new and important topics with the informants. The main question was: “How did you experience patients’ self-report questionnaires?” The follow-up questions addressed the difficulties experienced by patients in completing the questionnaires, problems with digital devices such as smartphones or tablets, and how self-reported data were used in interactions with patients during the treatment process. To ensure increased credibility and confirmability, the authors sought to clarify informants’ responses by discussing interpretations and asking follow-up questions during the interviews. All 19 therapists participated in focus group interviews. The focus group interviews were conducted between October and December 2022. No follow-up interviews were conducted with the therapist informants. The interviews were digitally recorded and transcribed verbatim by the second author (THM). The transcripts were not shared with the informants for comments or corrections. The average duration of the group interviews was 57 min.

All patients chose to be interviewed individually, and all patient interviews were conducted by the fourth author (HW). An interview guide developed by the authors (Supplementary Material, S2) was used in the patient interviews. The questions were open-ended, and the order was flexible, which allowed the researcher to explore important topics addressed by the informants. The main question was: “How did you experience answering questionnaires electronically”? The follow-up questions addressed difficulties with the use of digital devices, understanding the questionnaires, awareness of one’s own mental health challenges and the use of outcome measures in the treatment process. We did not conduct follow-up interviews with the informant patients. The six patients were interviewed in individual interviews between May and October 2023. The interviews were digitally recorded and transcribed verbatim by an external transcriber. The average duration of the individual interviews was 46 min.

### Procedure for collecting self-reported data

The study was conducted as part of the overarching project “Systematic Quality Assurance of Primary and Specialist Mental Health Care” [23]. This is a joint project comprising community and specialist mental healthcare, where electronic self-report questionnaires are implemented as part of routine clinical assessment. An overview of the questionnaires is shown in Table 3 and graphical illustrations of the web-based self-report portal are available in Supplementary Material, S3. The questionnaires included 24 questions covering sociodemographic information and 31 items from validated self-report questionnaires (see below). All patients received information through an online link provided by SMS, which directs respondents to a web-based portal containing information about the self-report assessment and the research project. When the self-report questionnaires are completed, patients are given the option to consent or decline to participate in the research. If the self-report forms are not completed within a week, a reminder is sent by SMS.

**Table 3:** Overview of proms and PREMs used in the study “Systematic quality assurance of primary and specialist mental health Care”

		PROM					PREM
Questionnaires		EQ-5D-5 L	GAD-7	PHQ-9	WSAS		SRS
Dimension/Items		5	7	9	5		4
Responses		5 categories	4-point Likert scale	4-point Likert scale	9-point Likert scale		1 category
Hash-marked		0-100					0–10

Note. EQ-5D-5 L = EuroQoL 5-Dimension 5-Level. GAD-7 = Generalized Anxiety Disorder 7-item Scale. PHQ-9 = Patient Health Questionnaire-9. WSAS = Work and Social Adjustment Scale. SRS = Session Rating Scale

To ensure that patients were provided with adequate guidance to patients when completing the questionnaires, the treatment facility in the project developed a new routine one year after the implementation of the PROMs/PREMs system. In addition to answering the questions at home, patients were offered the option to complete the questionnaires on a PC in the waiting room while waiting for their appointment. An employee was available to provide assistance and provided face-to-face information about the purpose of the PROMs. The patients spent 10–15 min on completing the questionnaires.

### Electronically administered PROMs/PREMs

#### PROMs

The EuroQoL 5-Dimension 5-Level questionnaire (EQ-5D-5 L) [32] is a five-item self-report instrument for the evaluation of health-related quality of life. It is based on a descriptive system that defines health in terms of five dimensions: mobility, self-care, usual activities, pain/discomfort and anxiety/depression. Each dimension has five response categories, ranging from no problems (e.g., “I have no problems in walking about”) to severe problems (e.g., “I am unable to walk about”). Respondents also rate their overall health on the day of the interview on a 0–100 hash-marked, vertical visual analogue scale (EQ-VAS). EQ-5D-5 L has good psychometric properties [33].

The Generalized Anxiety Disorder 7-item scale (GAD-7) [34] is a self-report questionnaire that measures symptoms of anxiety. The respondents rate symptoms (e.g., “Worrying too much about different things”) on a four-point Likert scale ranging from “Not at all” to “Nearly every day”. The severity levels range from mild (5–9), moderate (10–14) to severe (15–21). The GAD-7 has good psychometric properties and is useful for assessing general anxiety symptoms [35].

The Patient Health Questionnaire-9 (PHQ-9) [36] is a nine-item questionnaire that measures symptoms of depression (e.g., “Feeling down, depressed, or hopeless”) that are rated on a four-point Likert scale, ranging from “Not at all” to “Nearly every day”. Total scores range from 0 to 27, where a score of 5–9 indicates mild depression, 10–14 moderate depression, 15–19 moderately severe depression and 20–27 severe depression. The PHQ-9 has sound psychometric properties [37].

The Work and Social Adjustment Scale (WSAS) [38] is a five-item scale that assesses functional impairment in work ability, home management, social and leisure activities, and the maintenance of close relationships (e.g., “Because of my [disorder], my ability to work is impaired”). Items are rated on a scale ranging from 0 (“Not at all impaired”) to 8 (“Very severely impaired”). A total score below 10 indicates subclinical impairment, a score from 10 to 20 is significant impairment and a score above 20 suggests moderately or worse impairment. The WSAS has well-established psychometric properties [39].

#### PREMs

The Session Rating Scale (SRS) [40] is a four-item visual analogue scale that was developed to assess key dimensions of the therapeutic relationship, in accordance with Bordin’s [41] conceptualisation of the working alliance. The SRS is administered, scored and discussed at the end of each session to obtain real-time alliance feedback from patients so that alliance problems can be identified and addressed [40]. The SRS translates what is reported about the alliance into four visual analogue scales, to assess patients’ perceptions of respect and understanding, relevance of the goals and the topics, client–practitioner fit and overall alliance [42]. The SRS has acceptable psychometric properties [40].

### Data analysis

All the data were analysed using systematic text condensation (STC) in an iterative four-step process [43], which is a method that is suited for thematic cross-case analyses, inspired by Giorgi’s psychological phenomenology approach [44].

First, we analysed the transcripts of the therapist interviews, followed by the transcripts of the interviews with the patients. The analysis of the therapists’ interviews was conducted by the first (ER), second (THM), third (MSL) and sixth (AH) authors. The analysis began after the first focus group interview, for two reasons: (1) to make sure that the interview guide was comprehensive, and the questions well understood by the informants; and (2) to obtain a general impression and identify preliminary themes. The analyses continued simultaneously with the recruitment and interview processes until no new themes emerged from the transcripts. The data were considered saturated after the third focus group interview.

The analysis of the patient interviews began after the first three individual interviews had been conducted, for two reasons: (1) to ensure that the interview guide was thorough, and the questions were clearly understood; and (2) to capture a general overview and identify initial themes. The research team read the transcript manuscript from the first interview individually to attain a general perception and identify preliminary themes. The research team then read the transcript manuscript from the second and third interviews individually to identify preliminary themes, before discussing and sorting the preliminary themes.

Although the target was to recruit 10–15 patient informants, we were unable to recruit more than six. We made repeated attempts to encourage therapists to recruiting additional informants without success. The analysis continued until all six participants had been interviewed. Although the predefined recruitment target was not met, we estimate that the most important themes were covered, as not many new themes emerged in the fifth and sixth interviews.

All interviews were analysed following a four-step procedure: (1) total impression – from chaos to themes; (2) identifying and sorting meaning units – from themes to codes; (3) condensation – from codes to meaning; and (4) synthesising – from condensation to descriptions and concepts [43].

First, the transcribed interviews were read separately with an open mind to obtain a general impression and identify preliminary overarching themes. In the second step, the transcripts were systematically reviewed, line by line, to identify meaning units, and were classified and sorted into preliminary themes. The authors had several meetings to discuss and refine the subthemes and themes, and used individual Post-it notes in the process instead of software. The findings were first categorised into six and then four themes: notification of completion to patients, information, questionnaire and patients’ awareness. The latter theme was later refined to reflect the overall experiences and relationships between the therapists and patients. During the initial process from themes to codes, the researchers identified six themes and 31 codes from the therapists’ interviews.

For the patients’ interviews, seven themes and 27 codes were identified. Table 4 shows examples of themes and codes for both types of interviews. Table 5 gives examples of the process of categorising codes into meaning, with quotations from both therapists and patients.

**Table 4:** Examples of the process from themes to codes

**Therapist informants**				
Themes (Total 6)	Notification of completion to patients	Information	Questionnaire	Patients’ awareness
Codes (Total 31)	Language use, understanding and reflection. Many are tired of assessment and fail to answer. Conversation about why it is important to answer and what the data will be used for.	Routines for sending and reminding patients. About half of the patients have filled in the form in advance. A lot of information needs to be comprehended.	By answering the questionnaire, the patient assessment started earlier. The answers give us therapists better certainty about what services we can offer. What is important to the patient is important to us.	Patients’ participation – direct feedback from patients. Patients get ownership of the content of the questionnaires when they complete the form. The patients get an opportunity to reflect on their emotions when they complete the questionnaires.
**Patient informants**				
Themes (Total 7)	Therapist has empathy	Guidance by therapists	Insight into one’s own well-being	Easier to see how I’m doing
Codes (Total 27)	The way therapists talk to patients is important, the approach based on what the patient answers/needs. To be understood by a therapist is very satisfying.	It is good to receive follow-up/guidance when answering the questionnaires at the centre.	It is possible to see changes from session to session. The quality of life improves throughout treatment. Reporting in a questionnaire is easier than talking to a therapist.	Can show how we feel instead of telling. Makes it easier to put into words feelings/own experiences of mental health when using the tool.

**Table 5:** Examples of the condensation process – from code to meaning

Code	Meaning	Quotation (informants)
Technical guidance	*“It is difficult to fill in forms at home*,* when I don’t understand the meaning of the questions. I also think it is important to receive a reminder on my mobile phone that I have not filled in the form. It’s easy to forget*,* if I don’t fill in the form after the first reminder*.”	*“I struggle with concentration and then I need guidance.”*
Easier to show emotions using a form	*“I struggle to talk about feelings and how I feel*,* but by answering questions I can show it by answering the questionnaire*.”	*“It’s a useful tool that gives better insight into my mental well-being.”*
Therapist sees me	*“I find that the therapists are genuinely interested in helping me with my problems. Both in the first and subsequent consultations*,* they address my responses to the questionnaires I have completed*.”	*“It seems that the therapists are genuinely interested in helping.”*

In the third step, the meaning units within each subtheme, established in the second step of analysis, were reduced into a condensate – an artificial quotation maintaining, as far as possible, the original terminology applied by the informants. In the fourth step, the condensates of each subtheme were rewritten in general descriptions, and the final sorting of subthemes into the main themes was finalised. The analysis was further validated by a thorough review of the original transcripts of each interview to ensure that all points of significance were reflected in the results.

## Results

### Therapist informants

A total of 19 therapists were interviewed in three focus groups: six informants in group one (six women), six informants in group two (six women) and seven informants in group three (three men and four women). All interviews were conducted in a meeting room at the therapists’ workplace.

The findings were categorised into two themes: (1) a clear picture of patients’ challenges and (2) insufficient information, guidance and the purpose of patients’ self-report.

### A clear picture of patients’ challenges

The informants experienced that PROMs provided a clear picture of patients’ challenges, from the therapist’s perspective as well as from the patient’s perspective. When patients arrived for their first consultation, they had already formed a picture of what the challenge was and what goals they wanted to achieve to improve their life situation. Upon implementation of the PROMs, the therapists found that the patients had to reflect on their own situation and determine what was best for themselves.

Having patients complete the questionnaires themselves constitutes user-involvement, direct feedback from patient

The informants stated that they reviewed the self-reported responses together with the patient in the first consultation. They found that that it was easier to determine treatment goals, and they also felt that the patients had better awareness of what their health problems and challenges were. Thus, the therapists also found that the patients helped them to find the right treatment services that were appropriate for their individual challenges.

Mapping helps to find the right treatment and what kind of treatment the patients need

The therapists expressed that the data were “fresh”, as patients’ symptom pressure can fluctuate widely over the course of a few weeks. Furthermore, they reported that they always reviewed symptom scores of anxiety and depression to investigate whether a patient’s mental health varied over time and/or was triggered by specific situations.

The answers are fresh and situation-dependent, and we therefore need to have a follow-up conversation to check up on how they are doing

The therapists noted an increased awareness among the patients concerning their life situation after they had completed the questionnaires. They found that the patients gained a deeper understanding of their own health and their connection with daily activities. They said that this provided a common understanding between the therapist and the patient regarding what the challenges were and what services should be implemented.

The patient gains a better awareness of their own challenges when they have completed the questionnaires by themselves.

### Insufficient information, guidance and the purpose of self-reporting

Several of the therapists referred to the information letters that were sent to the patients. They stated that the letters contained a lot of information about the research itself and the importance of participating, but there was insufficient information about how completing the questionnaires might benefit the patients. The informants said that information about instructions and guidance for the patients on how to complete and understand the questionnaires was missing. They also felt that there was a lack of information regarding the importance of completing the forms and how the data would be used in the first consultation.

There is a lot of information about research and participation, but insufficient information for the patients about the purpose and how they should complete the forms

The therapists reported that approximately half of the patients had completed the self-report questionnaire before the first treatment session. One possible reason for this low figure may be that many patients have reading and writing difficulties, and others have major mental challenges. They also noted that patients with drug addiction problems sometimes lack phones, tablets, or PCs to answer questionnaires.

Many people do not respond to forms because they have reading and writing difficulties, and many have major mental health problems

The informants reported that most patients did not complete the questionnaires in advance. Thus, a new routine was implemented in the treatment service that ensured that patients were given guidance on how to complete the questionnaires. They said that they gave the patients oral information about the purpose of the use of self-reported data. The patients were provided with a tablet to complete the questionnaires upon arrival at the outpatient centre. Employees were available to answer patients’ questions about ambiguities in the questionnaire, and the first consultation was carried out immediately after they had completed the questionnaires. The therapists reported that they were able to adjust the treatment more quickly after they had reviewed the questionnaires together with the patients. After the new routine was implemented, the therapists found that all patients completed the questionnaires.

We have developed a routine that ensures that the patients are given guidance and information directly while answering five questionnaires

### Patient informants

A total of six patients (five men and one woman) were interviewed individually. All interviews were conducted in a meeting room at the outpatient centre and at KBT Vocational College.

The results were categorised into three themes: (1) needed guidance when answering the questionnaires; (2) answering the questionnaires provided better insight into mental health; and (3) therapist asked questions directly and listened.

#### Needed guidance when answering the questionnaires

Some patients said that it was difficult to complete forms when they were at home, as they struggled with concentration. Several reported that they waited to complete the form until they had an appointment at the outpatient centre, so that they could receive guidance by the therapist, and several others said that there were some questions that were difficult to understand.

One informant said that despite concentration difficulties, they were able to complete all the forms, before appointments with the therapist. The patients underlined the importance of receiving a reminder on their mobile phone if they had forgotten to complete the form. Most found that it was easier to complete a questionnaire electronically instead of on paper. They said that it was important that there were not too many questions and that it did not take too long to complete.

I struggle a bit with concentration, so it was important to have facilitation and guidance when completing the questionnaires at the outpatient centre

There were also some patients who said that they were able to answer the questions without guidance from therapists at the outpatient centre.

It was fine answering the questions at home and using my private computer

### Answering the questionnaire provided better insight into mental health

The informants said that they struggled to talk about their own feelings and how they felt, but by answering questions, they could express their feelings by ticking boxes on a form. They found answering the questionnaires easier than talking to a therapist about their feelings. They said that the questionnaires provided better insight into their mental health, and it was easier to see how things were when they saw the results of what they had filled in. The informants stated that it was much easier to see how they felt when they saw the curve of their scores rather than talking about their feelings. Many stated that they had great difficulty putting their own feelings into words.

All the patients felt that this tool was useful, as it provided insight into their mental health.

I struggle to talk about my own feelings, so it is easier to tick boxes on a form

It is a useful tool that provides better insight into my mental health

I see changes every time, and it appears as a curve – easy to see, and my life is better now than it was at the very beginning

#### The therapist asked questions directly and listened

The informants said that the therapists were genuinely interested in helping them with their problems. Both in the first session and later during treatment, the therapist reviewed their outcome measures and presented them with graphs of the scores. By discussing the results of the PROMs and PREMs with the therapist, the patients found that it was easy to see the curve showing symptom scores and their development in the treatment process.

Most of the patients said that in some weeks the scores on the self-report questionnaires indicated that there was good progress in the treatment process, whilst in other weeks there was a decline. They experienced that the therapists were good at asking questions and explore why elements comprising the “quality of life” were going well or badly. They also felt that the therapists were good at enquiring about their challenges, and they were very good at “reading them”.

The patients also felt that the therapists “understood them”, knew how to ask the right questions, and listened to them. A standard practice at the clinic is that patients may choose to go outside and talk with the therapist instead of staying in the office. The informants found that it was easier to walk and talk, as some struggled with concentration. They felt that therapists were skilled at asking them directly about everyday life and why they were doing well or badly. Because the therapists asked direct questions, the patients reported that they had to reflect on what they had done in treatment that might have contributed to their improvement, or whether there were aspects of the treatment plan they had not followed. Furthermore, patients felt that the therapists were skilled at documenting what they had talked about on a whiteboard. The informants said that they should only talk about two topics at each treatment appointment.

The therapist seems to be genuinely interested in helping

I am very pleased that the therapist listened to me and saw me and took me seriously

I felt that I had been seen, and they asked me direct questions

The patients found that the therapists had better information about their symptoms when they had been provided with answers from the questionnaire. Patients reported that during previous treatments, they had been asked about events during childhood and later in life, but now the questions were straight to the point. They said that they always reviewed the answers from the questionnaire and took the time they needed. The patients felt that they had a lot of time and that the therapists were flexible with the allotted time. They reported that they could call and arrange a new appointment if they were having a bad day, and they pointed out that they received treatment more often during bad periods. The patients felt that they were receiving skilled help and remarked that they were satisfied that both the therapist and the patient worked together with the self-reported data.

Before I got help here, I thought a lot about how to take my own life. After getting treatment, however, I don’t think that way anymore

## Discussion

In this study, we aimed to examine how therapists and patients experienced the implementation of electronic PROMs and PREMs in routine mental healthcare, and to identify the factors that promote positive experiences with care supported by PROMs and PREMs. The main finding of the study was that both therapists and patients experienced initial barriers, with a lack of information and guidance about answering questionnaires using smartphones, tablets or computers. After overcoming these barriers, the therapists reported that all patients completed PROMs/PREMs and stated that the patients had gained better insight into symptoms and challenges. This increased awareness led to the patients being more actively involved in the formulation of goals in the treatment process.

The patients experienced few difficulties answering questionnaires when they received guidance from the therapists. Several patients found it less demanding to answer questionnaires rather than talking about how they felt at that moment. They said that the tool provided better insights into their mental health, and it was easier to understand their current situation when they could see the results of the measures visualised in a graph. The use of graphs to illustrate the treatment progress was helpful, and the patients felt that the therapists were better informed about their symptoms after implementing the PROMs/PREMs system. The patients reported that they were satisfied with the therapists, as they asked direct questions about their adherence to the treatment programme since the previous consultation and actively listened to their current struggles in daily life. These findings illustrate how clinicians may use PROMs/PREMs actively throughout the course of treatment to improve patient engagement, which aligns well with previous research [45].

In accordance with the self-determination theory [46, 47], enhanced activation can strengthen patients’ sense of agency by fulfilling three core needs proposed by the theory – autonomy, competence and relatedness. Moreover, therapist guidance may boost patients’ feeling of competence, thereby promoting empowerment and fostering the individual’s intrinsic motivation for behavioural change.

### Implementing proms and PREMs – Do barriers still exist?

When selecting PROMs and PREMs, it is important to choose instruments that are both valid, sensitive to change and quick to use [9]. In the current study, we aimed to implement validated questionnaires with good psychometric properties but that were also brief, to ease the burden on respondents and prevent response fatigue. This mirrors the reasons for choosing instruments in previous studies [14]. The PROMs used included questionnaires assessing health-related quality of life (EQ-5D-5 L), anxiety symptoms (GAD-7), depressive symptoms (PHQ-9) and daily functioning (WSAS). A PREM that was used was the Session Rating Scale (SRS), relating to the working alliance. As demonstrated by the informants’ feedback, the measures were sensitive to change, as patients became more aware of session-to-session symptom changes and reported gaining more insight into their mental health. These results are in line with previous studies in Norwegian specialist mental healthcare that found patient feedback to improve service outcomes [30], and thus support the usefulness of also including routine patient feedback in Norwegian primary care. Moreover, this underscores the importance of continually reviewing the outcomes of PROMs/PREMs in close collaboration with the patient throughout treatment [45].

The implementation of a digital system for collecting PROMs/PREMS marks a shift from the traditional paper-based PROMs. The Technology Acceptance Model (TAM) is a framework for understanding how users accept new technologies, and emphasises that the adoption of technological systems largely depends on their ease of use and perceived usefulness [48]. Although the TAM has been less explored in mental healthcare, it provides valuable insights for addressing barriers encountered by both patients and clinicians. By examining how an intuitive interface and clear benefits shape user attitudes, the TAM highlights the importance of a positive user experience for improved willingness to apply digital tools. This understanding can guide the development of systems that not only minimise resistance but also actively foster patient engagement and integrated clinical practice.

The therapists found that the patients received insufficient information in the information letter about guidance and the purpose of the PROMs/PREMs system. This was in line with what the patients reported, as approximately half of the patients did not complete the questionnaire when the patient feedback system was implemented due to writing and reading difficulties. This barrier is similar to that identified in a review of the use of PROMs in routine cancer care, where patients were found to be incapable of completing PROMs for several reasons, including disability or difficulty reading and responding to the questions [22].

Successful implementation of PROMs and PREMs in clinical services requires flexible systems that can be adapted to patients’ and therapists’ needs [10]. After the implementation of the feedback system in the current study, we revised the routine for completing questionnaires by offering patients the option of completing them on site, so that employees could assist if needed. The therapists stated the importance of recency when completing questionnaires, as a patient’s symptom severity may fluctuate widely over the course of a few weeks. Following the revised routine for completing questionnaires at the service centre, all patients completed the self-report scales. The patients said that they were satisfied with the new routine, as they received immediate guidance from the therapists. This illustrates an important function of PROMs and PREMs to serve as an effective means of communication for patients to therapists [49].

A meta-review revealed that service providers reported that there was a lack of resources and that their skills and administration support were not evaluated in the implementation of PROMs [21]. In our study, we found the opposite, as the therapists reported that they were trained before and during the implementation of the PROMs/PREMs system in mental healthcare. The patients also stated that they were given enough time and resources in the treatment process, as they received more appointments with the therapists during periods of increased symptoms. This finding is in line with previous research and illustrates the importance of addressing potential barriers to implementation, such as sufficient training, regular feedback and follow-up assessments [21].

### A shared picture of patients’ challenges

The therapists reported that they obtained a clearer picture of patients’ challenges using PROMs and PREMs. They always reviewed the patient’s responses together with the patient and inquired about their answers, which is in line with previous studies [10, 50–52]. This is important, as patients may feel that their responses are not important if practitioners do not use the results from PROMs to inform treatment plans [53]. Patient-reported data should be the main source of information informing clinical decision-making, with clinician-reported data repositioned as secondary or adjunctive [4]. Because PROMs help to measure individual patient outcomes and can improve shared decision-making at the individual level, there is an ethical issue if the results from PROMs are not used by therapists. Hence, resources for implementing PROMs and PREMs focus on person-centred and value-based healthcare as these frameworks advocate consumer outcomes, values, preferences and perspectives [54, 55].

The therapists stated that patients experienced increased awareness of their difficulties, as the patients themselves highlighted the relationship between participation in daily activities and mood. However, an important aspect was that the patients said that they had reflected on their health and treatment process while they were answering the questionnaires. These findings correspond with self-determination theory, which describes how patients can be motivated in the treatment process by having increased knowledge about their mental health challenges [56].

Thornicroft and Slade [57] argue that the main limitation of PREMs is their tendency to reinforce a narrow view of recovery, especially for long-term service users who may experience “virtual institutionalisation”, where aspects of identity such as social networks, housing, and self-concept become indexed to mental illness. However, our findings suggest that such limitations may have been overcome in our study, where patient reported to have gained improved insight into their mental health. Many patients in our study expressed difficulty talking about feelings, but found it easier to communicate through questionnaires, and described this as less emotionally demanding. This aligns well with research emphasising the importance of the patient perspective, particularly in outcome-focused research [10, 50, 57]. It is possible that the therapists’ involvement in the preparation and implementation of PROMs and PREMs contributed to more positive evaluations. This supports the view that outcome measures – particularly those capturing direct health gains – may offer a more robust framework for evaluating mental health services.

Some implications for practice may be suggested based on the current study. Therapists reported that they gained an enhanced understanding of the patients’ problems, and the patients experienced that communicating difficult feelings through questionnaires was an important asset in therapy. By exploring these experiences, the findings of this study suggest that PROMs and PREMs could be implemented as part of routine community mental healthcare services. However, both the therapists and patients reported several barriers, such as the need for assistance when completing the self-report forms. This illustrates the importance of establishing flexible systems that may be easily adapted to the specific needs of the service recipients. Given the recent increase in referrals to mental healthcare, future studies should investigate how the successful implementation of PROMs and PREMs may contribute to more efficient resource allocation.

## Strengths and limitations

This study has several strengths. One strength is that both therapists and patients were interviewed, as this provided perspectives from both providers and patients on responding to electronic PROMs/PREMs. The nineteen therapists represented a variation in professions although not in gender. All therapists were trained in the use of the PROMs/PREMs system before and during implementation. Another strength is that all the patient interviews were conducted by staff from KBT Vocational College, who have extensive experience in interviewing patients with mental health challenges. One researcher had the main responsibility for all parts of the study and discussed all issues with the other researchers. Few studies have explored the implementation of PROMs/PREMs in community mental healthcare. This study provides new insights, as both therapists and patients experienced that the system provided a better understanding of the patients’ challenges.

However, some limitations must be kept in mind. The aim was to recruit 10–15 patient informants, but we were only able to recruit six persons. Although the reason for this is not known, this could indicate that some patients may have concerns about sharing their views on the topic. Moreover, it is possible that those who were recruited were atypical, and that informants who declined participation may have shared other experiences than those reported in the current study. The six informants were interviewed face-to-face, and in-depth interviews may provide more detailed information than group interviews. However, group interviews can also balance the opinions of others, leading to reflection and recalibration of their own statements. We aimed to build reflexivity, but the variation in background and experience may have influenced the study. It is a strength that both researchers and user representative experts conducted interviews, which allowed patient informants to be interviewed by persons who were experienced in interviewing user representatives and clinicians to be interviewed by researchers. However, it is possible that both the way in which the interviews were conducted and the thematic areas that were explored differed between the interviewers, depending on their background and experience. Some of the researchers participated as moderators in group interviews, and there is no doubt that the researchers may have influenced discussions and the topics that were explored [58]. Finally, it should also be noted that cultural aspects may have influenced the data collection and interpretation of the results, as has been suggested in previous studies on routine monitoring [27, 59].

## Conclusion

Overall, we found that PROMs and PREMs strengthened patients’ self-awareness of symptoms and challenges, which better enabled therapists to better provide person-centred services meeting the needs of the individual. However, many patients needed guidance when completing outcome measures, as some questions were hard to comprehend, and they had issues with concentration. The findings of this qualitative study highlight the importance of identifying and overcoming barriers when implementing PROMs and PREMs in a routine primary care setting.

## Supplementary Information

Below is the link to the electronic supplementary material.

Supplementary Material 1

## Data Availability

The data generated and analysed during this study are not publicly available due to concerns regarding participant privacy, but are available from the corresponding author upon reasonable request.

## Abbreviations

CFS: Clinical Feedback Systems
COREQ: Consolidated Criteria for Reporting Qualitative Studies
EQ-5D-5L: EuroQoL 5-Dimension 5-Level
GAD-7: Generalized Anxiety Disorder 7-item Scale
PHQ-9: Patient Health Questionnaire-9
PREM: Patient-Reported Experience Measure
PRM: Patient-Reported Measure
PROM: Patient-Reported Outcome Measure
SRS: Session Rating Scale
TAM: Technology Acceptance Model
WSAS: Work and Social Adjustment Scale
