# Supplementary material for: Therapists’ and patients’ experiences with electronic patient-reported outcome measures (PROMs) and patient-reported experience measures (PREMs) before and during treatment in community mental health services: a qualitative study in Norway
Source: BMC Psychiatry. 2025 Nov 13;25:1089. doi: 10.1186/s12888-025-07541-5 (PMC12613870; doi:10.1186/s12888-025-07541-5)

**Supplementary materials**

**S1.**

Interview guide – therapist informants

**Themes**

1. How did you experience implementing PROMS and PREMs and use of the outcomes from patient's?

- at what point do you go through the answers?
- the same day that the user has an appointment
- in a meeting with the patient
- I don't always have time
- what do you feel is the most useful for you? And why?
- do the answers provide better insight into what the patient's problems are?
- what was most useful - least useful for you?

1. Collaboration with patient

- What are your experiences with patients?
- Have the patients gained better insight into their own problems? In what way?
- Is it easier or more demanding to talk to the patient about the answers?
- Do the responses from the user have any significance for the content of the conversations? Or other offers?
- In what way do you prepare a meeting with the patient, based on the answers from the questionnaire?
- Is there a match between what the user perceives as problems and what you as the therapist perceive?
- What is done if there are major differences in understanding of what the problem is?
- Experiences without and with mapping questionnaire
- What is your experience with the use of submitted answers - does it result in a shorter course of treatment, a longer course, no change and why?
- Any other experiences you would like to share?
- can the use of data from mapping questionnaires be a useful tool for use at system level?

**S2.**

Interview guide - patient informants

**Themes**

1. How did you experience answering questionnaires electronically?

- In questionnaires on mobile, tablet or PC?
- How was it to read and understand the questions? (easy, medium, hard?)
- Have you completed such questionnaires on paper before? - if so, was it more difficult, just as easy or better (easier) with the use of a mobile phone, tablet or PC?
- What was better/more difficult?
- Did you get a reminder on your mobile about filling in the questionnaire?
- Did you complete the questionnaire right away? Or did you wait later?
- Were there any problems with ticking the answer options? Were the rubrics sufficiently large?
- Approximately how long did it take you (in total) to complete five questionnaires?

After completing the questionnaire - did you gain more insight or understanding of your problems?

- Can you say something about this?
- Do you think it was useful to fill in five questionnaires?
- What was most useful - least useful?

1. In a meeting with the therapist

- What experiences have you had in meeting with the therapist?
- Did the therapists go through the answers that you had completed in advance?
- Did you get a better understanding of your problems? Or was there no change?
- Did you change the assistance/other assistance measures after you and the therapist went through your answers?

1. Completing the questionnaire after treatment

What made you fill in the questionnaire when you received an SMS message?

- I want to participate in the study
- It gives me better insight into my own problems
- I think there was too much information and too many questions to answer
- It did not provide any new insight

Would you recommend such an arrangement to others - filling in a questionnaire before and after treatment?

Any other experiences you would like to share?

**S3.**

Illustration of the web-based self-report portal

**Figure S1.**

Example of Patient Health Questionnaire 9 items as presented to patients for self-report.


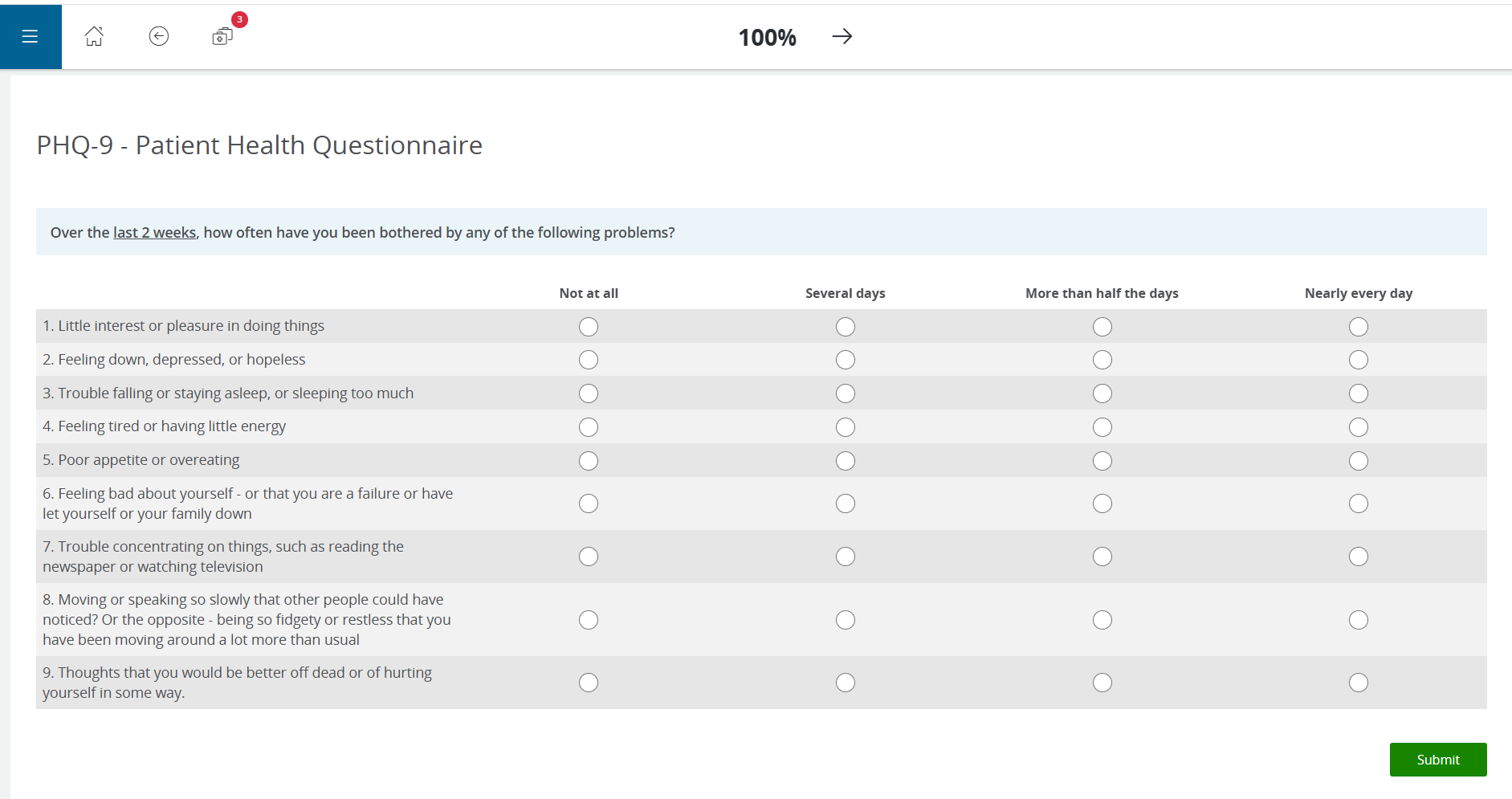


**Figure S2.**

Example of Patient Health Questionnaire 9 scores on two assessment points for a patient. Note: Fictitious data for illustration purposes.
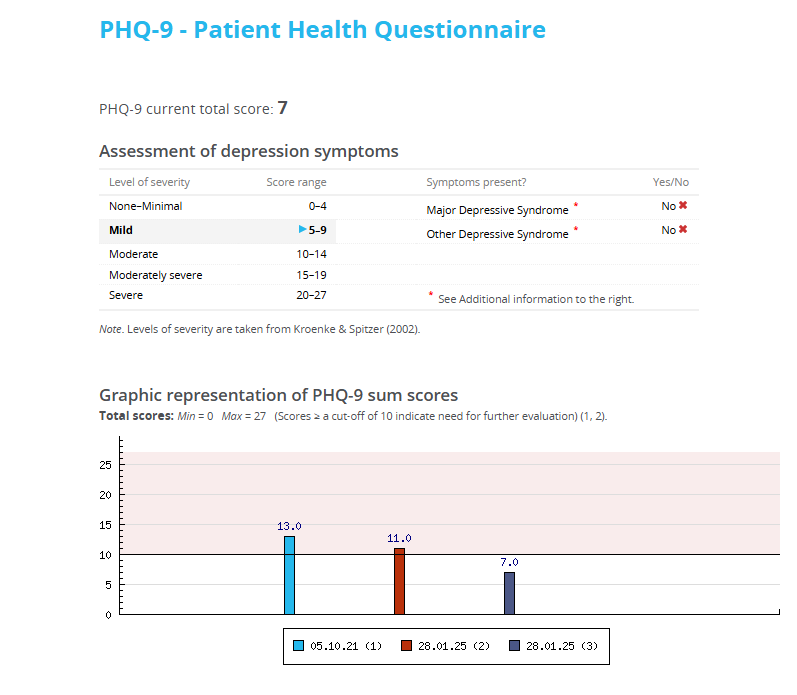

Supplement: Supplementary file 1 — Supplementary Material 1 [file 12888_2025_7541_MOESM1_ESM.docx]
